# Comorbidity Between Inflammatory Bowel Disease and Asthma and Allergic Diseases: A Genetically Informed Study

**DOI:** 10.1093/ibd/izae027

**Published:** 2024-02-27

**Authors:** Tong Gong, Bronwyn K Brew, Cecilia Lundholm, Awad I Smew, Arvid Harder, Ralf Kuja-Halkola, Jonas F Ludvigsson, Yi Lu, Catarina Almqvist

**Affiliations:** Department of Medical Epidemiology and Biostatistics, Karolinska Institutet, Stockholm, Sweden; Department of Medical Epidemiology and Biostatistics, Karolinska Institutet, Stockholm, Sweden; Centre for Big Data Research in Health and School of Clinical Medicine, University of New South Wales, Sydney, NSW, Australia; Department of Medical Epidemiology and Biostatistics, Karolinska Institutet, Stockholm, Sweden; Department of Medical Epidemiology and Biostatistics, Karolinska Institutet, Stockholm, Sweden; Department of Medical Epidemiology and Biostatistics, Karolinska Institutet, Stockholm, Sweden; Department of Medical Epidemiology and Biostatistics, Karolinska Institutet, Stockholm, Sweden; Department of Medical Epidemiology and Biostatistics, Karolinska Institutet, Stockholm, Sweden; Department of Pediatrics, Orebro University Hospital, Orebro, Sweden; Department of Medical Epidemiology and Biostatistics, Karolinska Institutet, Stockholm, Sweden; Department of Medical Epidemiology and Biostatistics, Karolinska Institutet, Stockholm, Sweden; Pediatric Allergy and Pulmonology Unit at Astrid Lindgren Children’s Hospital, Karolinska University Hospital, Stockholm, Sweden

**Keywords:** asthma, allergic rhinitis, eczema, Crohn’s disease, ulcerative colitis, familial co-aggregation, genetic correlation

## Abstract

**Background:**

Little is known about shared origins between inflammatory bowel disease (IBD) and allergic diseases (asthma, allergic rhinitis, and eczema). We aimed to expand current knowledge on the etiological sources of comorbidities between these disorders using a range of genetically informed methods.

**Methods:**

Within-individual and familial co-aggregation analysis was applied to 2 873 445 individuals born in Sweden from 1987 to 2014 and their first- and second-degree relatives. Quantitative genetic modeling was applied to 38 723 twin pairs to decompose the genetic and environmental sources for comorbidity. Polygenic risk score analysis between IBD and allergic diseases was conducted in 48 186 genotyped twins, and linkage disequilibrium score regression was applied using publicly available data to explore the genetic overlap.

**Results:**

IBD was associated with asthma (adjusted odds ratio [aOR], 1.35; 95% confidence interval [CI], 1.30 to 1.40), allergic rhinitis (aOR, 1.27; 95% CI, 1.20 to 1.34), and eczema (aOR, 1.47; 95% CI, 1.38 to 1.56), with similar estimates for ulcerative colitis or Crohn’s disease. The ORs for familial co-aggregation decreased with decreasing genetic relatedness. Quantitative genetic modeling revealed little evidence of common genetic factors between IBD and allergic diseases (eg, IBD and allergic rhinitis; genetic correlation r_a_ = 0.06; 95% CI, −0.03 to 0.15) but did reveal some evidence of unique environmental factors between IBD and eczema (r_e_ = 0.16; 95% CI, 0.00 to 0.32). Molecular genetic analyses were similarly null for IBD and allergic diseases, except for a slight association between Crohn’s disease polygenic risk score and eczema (OR, 1.09; 95% CI, 1.06 to 1.12).

**Conclusions:**

We found little evidence to support a shared origin between IBD and any allergic disease but weak evidence for shared genetic and unique environmental components for IBD and eczema.

**Key Messages**

**What is already known—**Previous studies have indicated comorbidity between inflammatory bowel disease (IBD) with asthma and allergic diseases.

**What is new here**—Using a range of genetically informed methods, we found little evidence of shared genetics between IBD and allergic diseases but some weak evidence for shared genetic and unique environmental components explaining the comorbidity of IBD and eczema.

**How can this study help patient care—**The findings help to improve the clinical understanding of the co-occurrence of inflammatory bowel diseases and allergic diseases.

## Introduction

Asthma and its genetically related allergic diseases, allergic rhinitis and eczema, are early childhood-onset atopic diseases characterized by a dysregulated adaptive immune response to environmental triggers together with chronic inflammation in the affected tissues.^1^ Inflammatory bowel disease (IBD) and its common subtypes—ulcerative colitis and Crohn’s disease—are lifelong systemic diseases that often demonstrate both gastrointestinal and extraintestinal symptoms.^2^ Although arising in different parts of the body— lung, skin, nasal mucosa, and gastrointestinal tract—these diseases may share pathogenesis through imbalance in microbiota composition, disruption of the epithelial barrier function, and/or a similar embryologic origin.

Epidemiological studies have consistently pointed to a high co-occurring prevalence (comorbidity) between asthma, allergic rhinitis, and eczema with IBD.^3-6^ Associations appear to be bidirectional, with no consistent pattern of whether allergic diseases may precede IBD or vice versa. Experimental studies have reported pathological processes that involve crosstalk between the gut and other mucosal tissues of the body such as the lung^7^ and the skin,^8^ suggesting a potential causal pathway between allergic diseases with IBD. Mendelian randomization studies have also addressed the issue of causality in an empirical way, using genetic variants as instrumental variables.^9-11^ However, results have not been consistent. Two studies investigating IBD and eczema, one using OpenGWAS data and the other using UK Biobank data, found contradictory results,^9,11^ and a Mendelian randomization study on IBD and asthma found that childhood-onset but not adulthood-onset asthma was inversely associated with IBD using the UK Biobank data.^10^ Therefore, obtaining reliable results from Mendelian randomization investigations remains challenging without summary data based on comparable sample sizes and well-defined phenotypes.

Alternative explanations to a causal hypothesis for high comorbidity rates include common risk factors—either shared genes or environment (eg, breastfeeding, smoking, antibiotics, microbiota).^12^ Allergic diseases and other inflammatory diseases of the gut such as gastroesophageal reflux^13^ and eosinophilic esophagitis^14^ may share genetic etiology. For example, the *GARP/LRRC32* gene, implicated from previous genome-wide association studies (GWASs) of asthma, eczema, IBD,^15^ and eosinophilic esophagitis,^16^ is reported to be involved in inflammatory cell function and activation.^17^ Furthermore, several genes commonly associated with asthma and IBD have been noted in the literature as possible candidates including interleukins (*IL1R*, *IL23R*), transmembrane proteins (*TLR4*), and tumor necrosis factor (*TL1A*).^12^ A meta-analysis of 2 GWASs on allergy (ie, self-reported allergy to cat/dust mite/pollen, positive skin prick test, or elevated IgE levels) and autoimmune diseases broadly (including IBD) found evidence for common genetic overlap between these disorders with single nucleotide polymorphisms (SNPs) common for immune pathways and chromatin accessible in immune cells.^18^ This would support a common genetic origin for allergic and autoimmune diseases.

**Figure 1.**
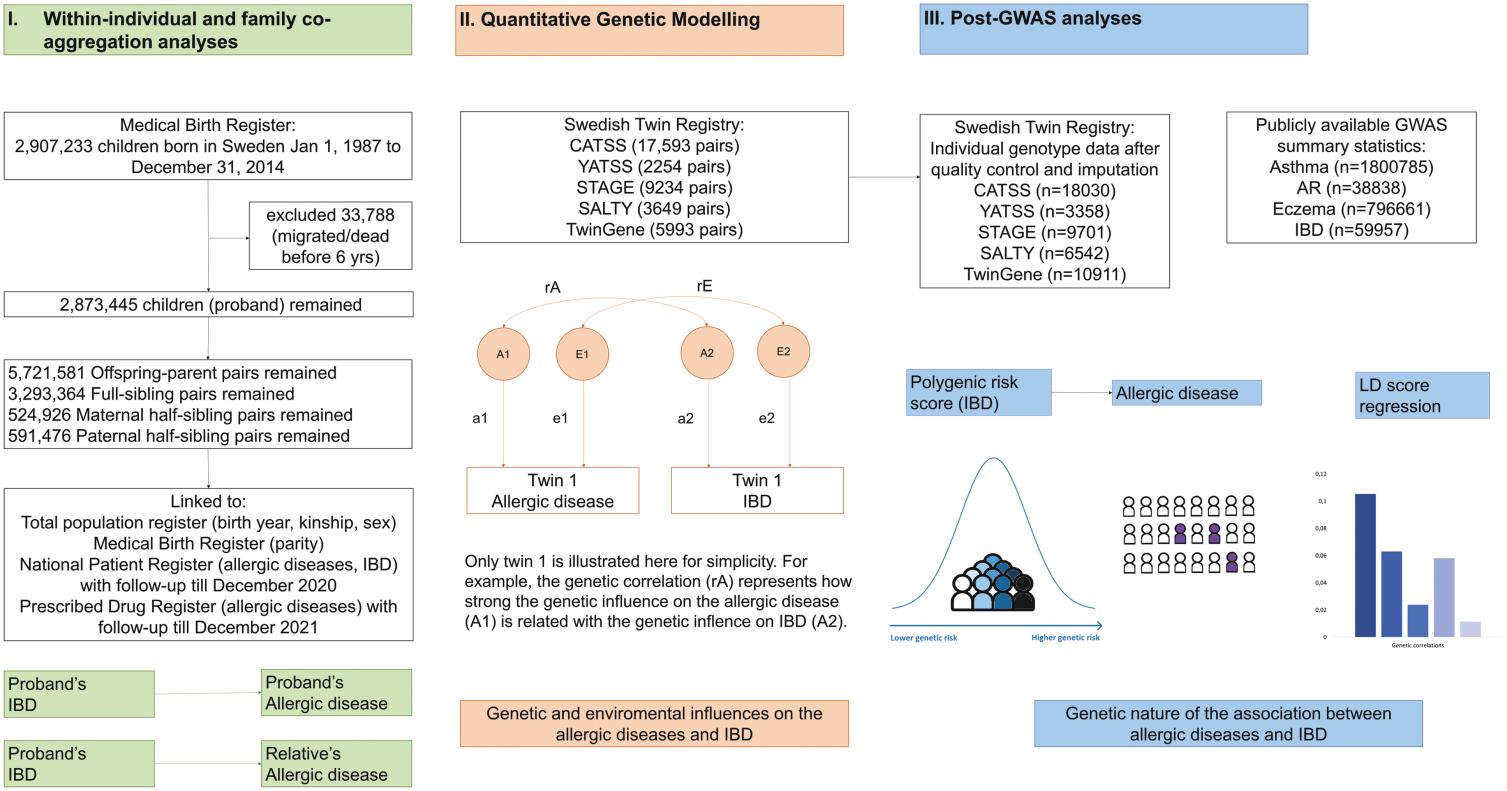
Overview of the study. AR, allergic rhinitis; CATSS, Child and Adolescent Twin Study of Sweden; GWAS, genome-wide association study; IBD, inflammatory bowel disease; LD, linkage disequilibrium; SALTY, Screening Across the Lifespan Twin study in Younger adults; STAGE, Study of Twin Adults: Genes and Environment; YATSS, Young Adult Twins in Sweden Study.

Population-level multigenerational studies such as familial aggregation and classical twin analyses can provide epidemiological evidence for a shared genetic or environmental origin of allergic diseases and IBD. An earlier study by Hemminki et al^19^ used familial aggregation methods with siblings and parents to show evidence for a familial association between asthma with ulcerative colitis and Crohn’s disease but was limited to inpatient diagnostic records. In the current study, we extend the definition of asthma to include outpatient care and medication data, include allergic rhinitis and eczema to the analysis, expand the number of observed familial relationship types, and add quantitative genetic modeling. In addition, we use recently published summary-level data and individual genetic data (see Figure 1) to perform post-GWAS analyses including polygenic risk score (PRS) analysis and linkage disequilibrium score (LDSC) regression to attempt to further triangulate evidence for a shared genetic origin between asthma, allergic rhinitis, and eczema with IBD.

## Methods

### Study Populations and Data Sources

#### Population-based cohort

The population-based cohort included all individuals (N = 2 907 237, referred to as probands) born in Sweden between January 1, 1987, and December 31, 2014, identified from the Medical Birth Register (Figure 1). Probands were then linked to records from other national registers including the Total Population Register, the National Patient Register (inpatient care data from 1987/outpatient care data 2001 to February 2022),^20^ and the Swedish Prescribed Drug Register^21^ (data available July 2005 to February 2022) to retrieve information on first-degree relatives (ie, parent, full siblings) and second-degree relatives (ie, maternal and paternal half-siblings), diseases of interest, and death. Probands and their first-degree (ie, full siblings) and second-degree relatives who emigrated from Sweden, died before 6 years of age, or who were born before 1987 or after 2014 were excluded from the cohort. Calendar year of birth, parity, sex of probands, and identification of relatives were retrieved from the Medical Birth Register and the Multi-Generation Register, which is a part of the Total Population Register.^22^

#### Twin subcohorts

National health register information (until 2016) was linked to 5 subcohorts of child and adult twins (total twin pairs: n = 38 723) from the Swedish Twin Registry including: 17 593 pairs from the Child and Adolescent Twin Study of Sweden (CATSS), born 1992 to 2012; 2254 pairs from the Young Adult Twins in Sweden Study (YATSS), born 1985 to 1991; 9234 pairs from the Study of Twin Adults: Genes and Environment (STAGE), born 1958 to 1985; 3649 pairs from the Screening Across the Lifespan Twin study in Younger adults (SALTY), born 1943 to 1958; and 5993 pairs from TwinGene, born 1911 to 1958 (Figure 1).^23^ Twins were excluded if they had incomplete phenotypic information or did not give consent to register linkage. Information on zygosity was retrieved from either the collected DNA sample or the answers to 5 questions on twin similarity throughout questionnaires/interviews (with a ≥95% probability of correct categorization).

DNA for PRS analyses were obtained at the study enrollment from saliva samples for CATSS (including 2 waves of data collection), YATSS, STAGE, and SALTY and from peripheral blood samples for TwinGene. Individuals with DNA samples were genotyped using the Illumina PsychArray BeadChip (for CATSS wave 1, SALTY), 650K Illumina Global Screening Array BeadChip (for CATSS wave 2, YATSS, and STAGE), and the Illumina OmniExpress BeadChip (for TwinGene). Standard quality control and imputation procedures were performed (see detailed information from Supplementary Materials). After removing low-quality samples and imputing the genotypes of monozygotic (MZ) co-twins from their paired genotyped twins, we had 48 542 unique samples (13 206 from CATSS wave 1, 4824 from CATSS wave 2, 3358 from YATSS, 9701 twins from STAGE, 6542 twins from SALTY, 10 911 twins from TwinGene) (Figure 1).

### Asthma, Allergic Rhinitis, Eczema, and IBD

We defined individuals with asthma as those who met either of the following criteria: (1) any diagnostic records of asthma from the National Patient Register or (2) at least 2 asthma-relevant medications from the Swedish Prescribed Drug Register, based on a previously validated algorithm (see Supplementary Table 1 for detailed information on included medications).^24^ Allergic rhinitis and eczema were defined based on algorithms similarly incorporating hospital diagnostic and medication criteria by Henriksen et al (see Supplementary Table 1).^25^ Furthermore, we categorized IBD into 2 main subtypes, ulcerative colitis and Crohn’s disease, based on at least 2 healthcare visits with relevant diagnostic codes (International Classification of Diseases–Eighth/Ninth/Tenth Revision codes 563.10, 569.02/556/K51 for ulcerative colitis and 563.00/555/K50 for Crohn’s disease).^26^ We also included a broader definition for IBD, which included having ≥2 diagnoses of ulcerative colitis, Crohn’s disease, or unspecified IBD (International Classification of Diseases–Eighth Revision codes 563.98-563.99) reported in the National Patient Register (ie, allowing individuals to have different diagnostic codes at different healthcare visits anytime during follow-up) (see Supplementary Table 1).

### Polygenic Risk Scores

PRSs for IBD, asthma, allergic rhinitis, and eczema were generated in each subcohort of twins (as separate target sets), using summary statistic data from meta-analyses of the largest publicly available GWAS on the relevant traits to date. We conducted searches of GWASs using the GWAS Catalog and medRxiv and selected based on sample size with European ancestry and exclusion of the Swedish twin samples (detailed information on the summary data search and about the discovery sets can be found in Supplementary Methods and Supplementary Table 2).

We used the SBayesR method to generate individual PRSs for each trait. The method has demonstrated better prediction accuracy compared with other conventional PRS approaches such as clumping and thresholding.^27^ Due to the complex nature of linkage disequilibrium, it is recommended to exclude the major histocompatibility complex (MHC) region when using SBayesR. However, some of the traits we studied have significant variants in the MHC region. Therefore, we first extracted the most significant variant in the MHC region for each trait and applied SBayesR as normal (which excludes the MHC region). Then, we added the significant variant back in with the raw effect size from the original GWAS. Last, we generated the PRS using the plink2’s -score command and standardized the scores.

### Statistical Analysis

#### Within-individual association and familial co-aggregation

Using the population-based cohort, within-individual associations between IBD/ulcerative colitis/Crohn’s disease and allergic diseases (ie, asthma, allergic rhinitis, and eczema) were estimated using logistic regression for probands with IBD/ulcerative colitis/Crohn’s disease vs probands without. Familial co-aggregation between IBD and allergic diseases was estimated for probands and relatives with first degree (full siblings, parent-proband) and second degree (half-siblings—either maternal or paternal) of relatedness for probands with IBD/ulcerative colitis/Crohn’s disease vs probands without. Estimates were reported from (1) crude models, (2) models adjusted for potential confounders (ie, calendar year of birth, parity, sex for both proband and relatives [note: parity variable was not available for parents]), and (3) models additionally adjusted for IBD/ulcerative colitis/Crohn’s disease in the first- and second-degree relatives. In families with multiple siblings sharing the same parents, all possible pairs for analysis were kept, and a cluster robust sandwich estimator for standard errors was used to correct for familial clustering. The analyses were performed in Stata version 15.1 (StataCorp).

#### Quantitative genetic modeling

Univariate and bivariate quantitative genetic analyses of the twin subcohorts were used to decompose the phenotypic variation of each allergic disease and IBD. Unfortunately, it was not possible to decompose ulcerative colitis and Crohn’s disease separately due to the limited number of cases. First, individual cross-trait (phenotypic), cross-twin within-trait, and cross-twin cross-trait phenotypic correlations were estimated as tetrachoric correlations for each of the allergic diseases with IBD. Second, univariate and bivariate liability threshold structural equation models were fitted to quantify the proportion of variation in liability to allergic diseases and IBD that was due to genetic and environmental variation.^28^ The fraction of variance and covariance in liability explained was estimated for the following components: (1) additive genetic effects (noted as A, assuming monozygotic [MZ] twins sharing 100% and dizygotic [DZ] twins sharing 50% of genetic variance); (2a) nonadditive/dominant genetic effects (noted as D, assuming MZ twins sharing 100% and DZ twins sharing 25% of the variance due to dominance deviations from the additive effects) or (2b) shared environmental effects (environmental influences which MZ and DZ twins share 100%, noted as C); and (3) unique environmental effects (environmental influences unique to each individual with measurement error included, noted as E).^29^ We fitted univariate ACE, ADE, and AE models for asthma, allergic rhinitis, eczema, and IBD separately, with adjustment for sex and birth year. Then we fitted bivariate ACE, ADE, and AE models for each allergic disease with IBD adjusting for sex and birth year. For simplified interpretation of bivariate ADE models, the broad-sense heritability (H = A + D) was presented. Akaike information criterion and likelihood ratio test results were used to select the best-fitting models. The Wald method was used to calculate 95% confidence intervals (CIs) for all parameter estimates. All analyses were performed using R software (version 3.6.2; R Foundation for Statistical Computing) with the OpenMx package (version 2.14.1).

#### PRS analysis

First, we correlated the PRS of each trait with the relevant phenotypic measures to confirm whether the PRS had power in the target sets. Second, we used generalized estimating equations (logit link function) with robust variance estimators to model the PRS of one disease (eg, IBD) as a predictor of the other disease phenotype (eg, asthma) while accounting for the twin clustering. Because the PRS estimates were standardized, we combined data of all target sets and presented model estimates adjusted for birth year and sex, as well as the interaction term of the subcohort and top 5 principal components (n = 48 186), which control for population stratification of each subcohort. The analyses were performed in Stata version 15.1.

#### LDSC regression

The genetic overlap between IBD and allergic diseases was estimated using published GWAS summary statistics for LDSC regression analysis (see Supplementary Table 2). Association test statistics (*z* scores, beta coefficients, or odds ratios) were regressed on their linkage disequilibrium scores. The SNP-based heritability (h^2^_SNP_ converted to liability scale with population-based prevalence from literature and sample prevalence data from summary statistics)^30-33^ was estimated and genetic correlation (r_g_) of each allergic disease with IBD using LDSC after Bonferroni correction was calculated.^34^ Analysis was performed using LDSC tool (Python 2.7.5; Python Software Foundation).

### Ethical Considerations

The current study was approved by the Swedish Ethical Review Authority, and informed consents were received from all twin participants and waived for the others. All data were pseudonymized prior to analyses.

## Results

Characteristics of the population-based cohort of probands born in Sweden from 1987 to 2014 and relatives are presented in Supplementary Table 3. The prevalence of IBD, ulcerative colitis, and Crohn’s disease in the proband population were 0.7%, 0.3%, and 0.3%, respectively. The prevalence of asthma, allergic rhinitis, and eczema in the proband population were 18.0%, 30.6%, and 16.3%, respectively. Prevalence for all diseases in full siblings and half-siblings were similar to probands, and IBD and ulcerative colitis were slightly raised in parents (1.0% and 0.6%, respectively), whereas asthma prevalence was slightly lower (13.4%).

### Within-Individual Association and Familial Co-Aggregation

**Figure 2.**
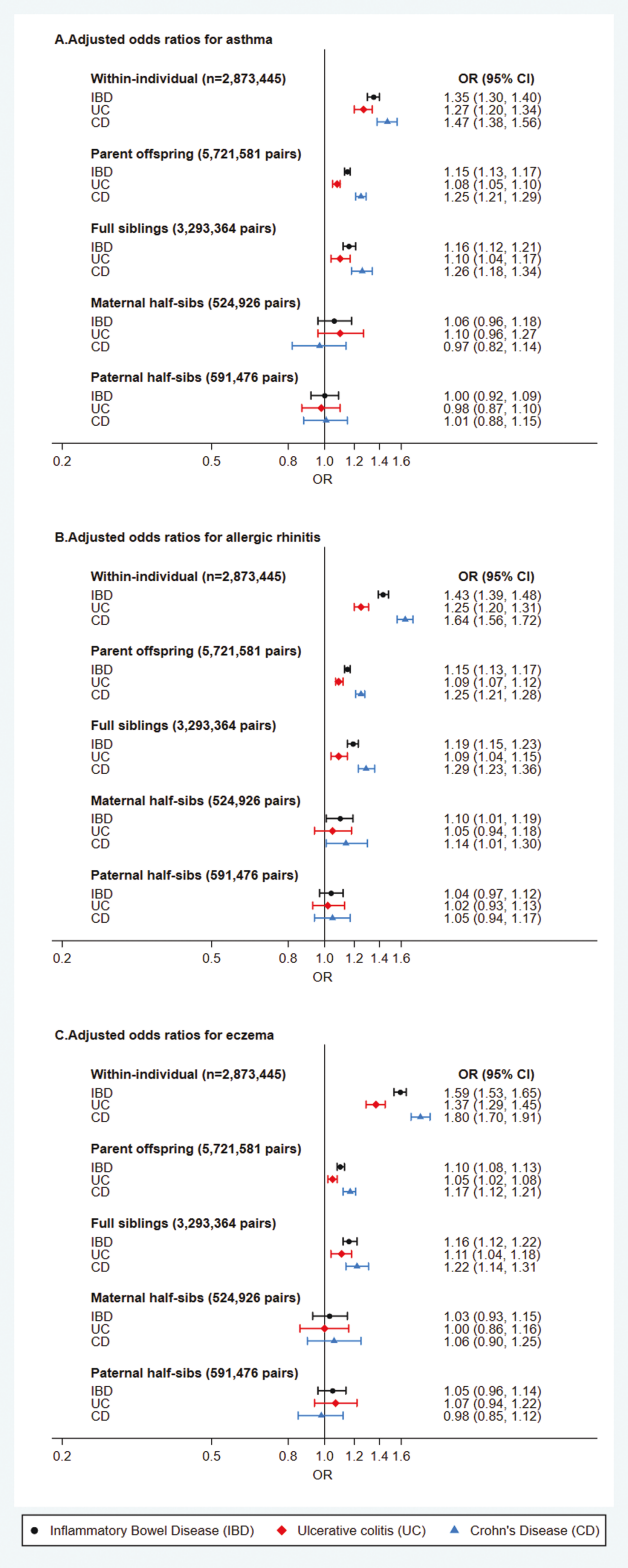
Within-individual association and family co-aggregation of atopic diseases and inflammatory bowel disease (IBD). The presented estimates are based on models adjusted for birth year, sex, and parity. CD, Crohn’s disease; CI, confidence interval; OR, odds ratio; UC, ulcerative colitis.

In the population-based cohort, we observed increased risk of having asthma, allergic rhinitis, and eczema within individuals (probands) that had IBD after adjusting for sex, parity, and birth year: adjusted odds ratios (aORs) of 1.35 (95% CI, 1.30-1.40), 1.27 (95% CI, 1.20-1.34), and 1.47 (95% CI, 1.38-1.56), respectively (Figure 2; Supplementary Table 4). ORs were slightly higher for Crohn’s disease than ulcerative colitis with each allergic disease. Familial co-aggregation showed a decrease in associations toward the null in a stepwise fashion with decreasing relatedness (genetic and shared environment), suggesting a shared origin between IBD and allergic diseases (Figure 2). For first-degree relatives (full sibling and parents), all effect sizes between allergic diseases and IBD/ulcerative colitis/Crohn’s disease were lower than the within-individual results, while associations for second-degree relatives (half-siblings) were all close to null (Figure 2; Supplementary Table 4). For example, the association between asthma and Crohn’s disease within individual had an aOR of 1.47 (95% CI, 1.38-1.56), between probands (IBD) and full siblings (Crohn’s disease) had an aOR of 1.25 (95% CI, 1.17-1.33), between probands (IBD) and parents (Crohn’s disease) had an aOR of 1.25 (95% CI, 1.20-1.29), and between probands (IBD) and half-siblings (Crohn’s disease) had a maternal aOR of 0.97 (95% CI, 0.82-1.14) and a paternal aOR of 1.01 (95% CI, 0.88-1.15).

Using the twin subcohorts, we observed that ORs of MZ twins having asthma, allergic rhinitis, or eczema when their co-twin had IBD were mostly above 1, although with wide CIs (Supplementary Table 4). ORs again were slightly higher for Crohn’s disease than ulcerative colitis. However, no clear associations were seen among DZ twins for the co-aggregation of allergic diseases and IBD (Supplementary Table 4).

### Quantitative Genetic Modeling

Univariate results of the twin subcohorts found higher correlations for each allergic disease or IBD in MZ twins compared with DZ twins (Table 1), suggesting genetic heritability for each disease. Sex-specific correlations for each allergic disease and IBD were similar between males and females (Supplementary Table 5). Univariate quantitative genetic modeling identified AE as the best-fitting model (with lowest Akaike information criterion) for most diseases and suggested a minimal role of dominant genetic or shared environmental influences. In the AE model, additive genetic components explained 75% of the variance in liability for IBD (95% CI, 0.68-0.82), 53% variance for allergic rhinitis (95% CI, 0.51-0.55), and 44% variance for eczema (95% CI, 0.41-0.47). However, the ACE model fitted asthma best, with 66% of the variance explained by additive genetic components (95% CI, 0.59-0.72), 6% explained by shared environment (95% CI, 0.00-0.11), and 28% explained by unique environment (95% CI, 0.26-0.31) (Table 1).

**Table 1.** Univariate twin correlations (within-trait cross-twin) and univariate twin model parameter estimates for IBD and allergic diseases in all 5 twin subcohorts combined (MZ twin pairs: n = 13 028, DZ twin pairs: n = 25 605)

	Twin correlations (95%CI)		Model	Twin model estimates (95% CI)^a^				
	r_MZ_	r_DZ_		a^2^	c^2^ or d^2^	h^2^	e^2^	AIC
IBD	0.72 (0.66 to 0.77)	0.46 (0.39 to 0.52)	ACE ADE AE^b^	0.54 (0.30 to 0.79) 0.75 (0.68 to 0.82) 0.75 (0.68 to 0.82)^b^	0.18 (−0.01 to 0.38) 0.00 (0.00 to 0.00) —	— 0.75 (0.68 to 0.82) —	0.27 (0.19 to 0.35) 0.25 (0.18 to 0.32) 0.25 (0.18 to 0.32)^b^	5037.76 5039.08 5037.08^b^
Asthma	0.71 (0.69 to 0.72)	0.39 (0.37 to 0.40)	ACE^b^ ADE AE	0.66 (0.59 to 0.72)^b^ 0.72 (0.70 to 0.74) 0.72 (0.70 to 0.74)	0.06 (0.00 to 0.11)^b^ 0.00 (0.00 to 0.00) —	— 0.66 (0.59 to 0.72) —	0.28 (0.26 to 0.31)^b^ 0.28 (0.26 to 0.30) 0.28 (0.26 to 0.30)	26 866.73^b^ 26 869.75 26 867.75
Allergic rhinitis	0.52 (0.50 to 0.54)	0.28 (0.26 to 0.29)	ACE ADE AE^b^	0.49 (0.42 to 0.56) 0.53 (0.51 to 0.55) 0.53 (0.51 to 0.55)^b^	0.03 (−0.02 to 0.09) 0.00 (0.00 to 0.00) —	— 0.53 (0.51 to 0.55) —	0.48 (0.45 to 0.51) 0.47 (0.45 to 0.49) 0.47 (0.45 to 0.49)^b^	36 249.36 36 250.61 36 248.61^b^
Eczema	0.43 (0.40 to 0.46)	0.23 (0.21 to 0.25)	ACE ADE AE^b^	0.43 (0.34 to 0.53) 0.44 (0.41 to 0.47) 0.44 (0.41 to 0.47)^b^	0.01 (−0.06 to 0.08) 0.00 (0.00 to 0.00) —	— 0.44 (0.41 to 0.47) —	0.56 (0.52 to 0.60) 0.56 (0.53 to 0.59) 0.56 (0.53 to 0.59)^b^	28 461.62 28 462.43 28 460.63^b^

Abbreviations: a^2^, additive genetic component; AIC, Akaike information criterion; c^2^, shared environmental component; CI, confidence interval; d^2^, nonadditive/dominant genetic component; DZ, dizygotic; e^2^, nonshared environmental component (including measurement errors); h^2^, broad-sense heritability component (a^2^ + d^2^); IBD, inflammatory bowel disease; MZ, monozygotic.

^a^All models were adjusted for sex and birth year (continuous, standardized).

^b^Best fitting models and estimates.

The phenotypic bivariate correlations (within individual) between IBD and allergic diseases were weak for IBD with asthma (r = 0.05; 95% CI 0.01-0.09), allergic rhinitis (r = 0.07; 95% CI 0.03-0.11), and eczema (r = 0.11; 95% CI 0.07-0.15). Cross-twin cross-trait correlations were also weak but were higher for MZ twins than DZ twins for asthma and eczema with IBD, suggesting weak genetic influences (Table 2). We also found similar results for male and female MZ and DZ twins (Supplementary Table 5). In bivariate quantitative genetic modeling, the AE model fitted best for all combinations of IBD with allergic diseases (Supplementary Tables 6-8). Genetic correlations (r_a_) were low for IBD with each allergic disease, and CIs crossed the null (Table 2). Unique environment correlations (r_e_) were slightly higher, but only IBD and eczema were statistically significant (r_e_ = 0.16; 95% CI, 0.00-0.32) (Table 2).

**Table 2.** Bivariate cross-twin cross-trait correlations and AE bivariate quantitative genetic model estimates for IBD and allergic diseases in all 5 twin subcohorts combined.

	Phenotypic correlations	Cross-twin cross-trait correlations (95%CI)		Twin model estimates (95% CI)^a^	
		r_MZ_	r_DZ_	r_a_	r_e_
**IBD and asthma**	0.05 (0.01 to 0.09)	0.06 (−0.02 to 0.14)	−0.06 (−0.12 to 0.00)	0.05 (−0.03 to 0.12)	0.08 (−0.10 to 0.27)
**IBD and allergic rhinitis**	0.07 (0.03 to 0.11)	0.04 (−0.03 to 0.11)	0.02 (−0.03 to 0.06)	0.06 (−0.03 to 0.15)	0.12 (−0.04 to 0.29)
**IBD and eczema**	0.11 (0.07 to 0.15)	0.09 (0.01 to 0.17)	−0.03 (−0.09 to 0.02)	0.06 (−0.05 to 0.17)	0.16 (0.00 to 0.32)

Abbreviations: a, additive genetic component; CI, confidence interval; e, nonshared environmental component (including measurement errors); IBD, inflammatory bowel disease.

^a^All models were adjusted for sex and birth year (continuous, standardized).

### PRS Analysis

**Figure 3.**
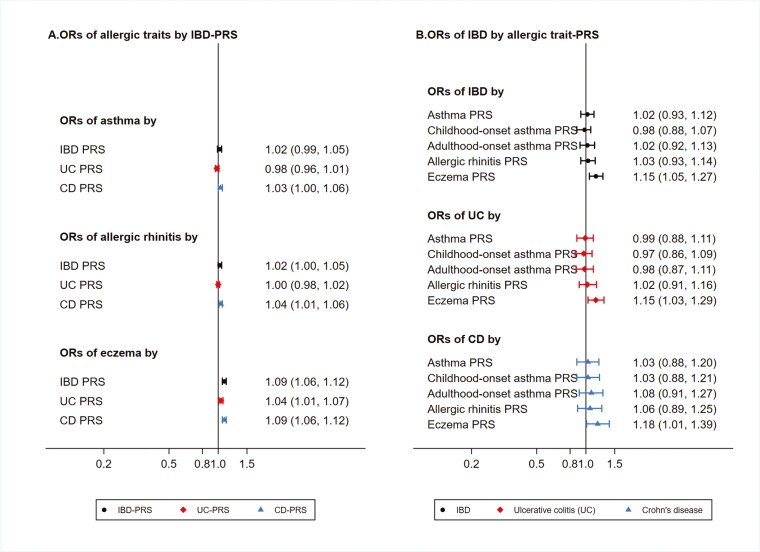
Associations (odds ratios [ORs] and 95% confidence intervals [CIs]) of polygenic risk scores (PRSs) and phenotypes between inflammatory bowel disease (IBD) and allergic diseases among 48 186 twins with genotype data. CD, Crohn’s disease; UC, ulcerative colitis.

Among the genotyped twins, we observed that PRS of asthma and IBD had a better predictive power than the allergic rhinitis and eczema PRSs in each twin subcohort (Supplementary Table 9 and Supplementary Figure 1). Most of the associations between IBD-PRS and each allergic disease were close to 1 (Figure 3; Supplementary Table 10), except for a pooled weak association between IBD-PRS and risk of eczema (for per-SD increase in IBD-PRS: OR, 1.09; 95% CI, 1.06-1.12) (Figure 3). When broken down into subtypes of IBD, associations with eczema were slightly higher for Crohn’s disease and PRS than ulcerative colitis and PRS (Supplementary Table 10). There were no statistically significant association between the IBD phenotype and the allergic disease and PRSs, including a subanalysis by age of onset of asthma, except that the association between eczema-PRS and IBD was equivalent to what we observed between IBD-PRS and eczema, albeit with wider CIs (Figure 3; Supplementary Table 11).

### Linkage Disequilibrium Score Regression

Low-to-moderate SNP-based heritability for each allergic disease and IBD was observed (h^2^_SNP_ = 0.08-0.33) (Table 3). There were no statistically significant genetic correlations observed between any of the allergic diseases and IBD/ulcerative colitis/Crohn’s disease except for weak genetic correlations between asthma with IBD and Crohn’s disease, which was not statistically significant after Bonferroni correction (*P* value threshold = .01 and .005 after correcting for 5 tests [IBD] and 10 tests [ulcerative colitis/Crohn’s disease], respectively): r_g_ = 0.11, *P* values = .01 (Table 3; Supplementary Table 12).

**Table 3.** Linkage-disequilibrium regression results between IBD and asthma, allergic rhinitis, and eczema.

Allergic disease	n for allergic disease (sample prevalence, population prevalence)^a^	h^2^_SNP (allergic disease)_ (SE)^b^ ¤	Genetic correlation with IBD, r_g_ (95% CI)	*P* value^c^
Asthma	1 800 785 (8.5%, 8%)	0.079 (0.005)	0.105 (0.021 to 0.190)	.0145
Adulthood-onset asthma	327 253 (8.1%, 8%)	0.126 (0.010)	0.063 (−0.027 to 0.153)	.1703
Childhood-onset asthma	314 633 (4.4%, 5%)	0.298 (0.031)	0.024 (−0.080 to 0.127)	.6527
Allergic rhinitis	38 838 (27.2%, 25%)	0.119 (0.024)	0.058 (−0.078 to 0.194)	.4026
Eczema	796 661 (2.8%, 3%)	0.078 (0.015)	0.011 (−0.026 to 0.048)	.546

Abbreviations: CI, confidence interval; GWAS, genome-wide association study; IBD, inflammatory bowel disease; SNP, single nucleotide polymorphism.

^a^The population prevalence estimates were based on literature for most traits. However, we assumed a lower population prevalence of childhood-onset asthma to match the reported SNP-based heritability from the original GWAS.

^b^The SNP-based heritability for IBD, ie, h^2^_SNP (IBD)_ is 0.168 (0.015) based on the population prevalence at 1% for IBD.

^c^Presented based on individual analysis before Bonferroni correction. The Bonferroni-corrected significance across 5 tested linkage disequilibrium scores for genetic correlation should be .01.

## Discussion

In this study, which used several population-based samples and employed 4 different genetically informed analysis methods, we found minimal to no evidence for a genetic correlation between IBD and each of the allergic diseases—asthma, allergic rhinitis, and eczema. First, the familial co-aggregation analysis suggested a modest familial association between IBD and each allergic disease, particularly for Crohn’s disease, which either implies a genetic or shared environmental cause of comorbidity. Second, the quantitative genetic modeling showed that although IBD and each allergic disease individually exhibited a moderate-to-high heritability, there was a weak to negligible genetic correlation between IBD and each allergic disease. Furthermore, there was suggestive evidence for a unique environmental component explaining the comorbidity of IBD and eczema. Last, using PRS and LDSC analyses, there was no compelling evidence for a genetic overlap between IBD and allergic diseases except for a possible weak bidirectional association between IBD and eczema.

There has been considerable interest in the relationship between asthma and other comorbid immune-mediated conditions, whereas there has been less interest for IBD.^35,36^ With the fundamental link to atopy between asthma, allergic rhinitis and eczema, this study comprehensively investigated the association between these 3 common allergic conditions and IBD within individuals and families. We showed approximately 18% prevalence of asthma, 31% allergic rhinitis, 16% eczema, and 0.7% IBD, which is slightly above or aligns closely with the numbers reported in previous population-based cohort studies from Sweden.^25,33,37,38^ IBD subtypes and asthma measures have in previous validation studies shown minimal misclassification (ie, ≥90% and ~80% positive predictive values, respectively).^24,26^ The milder allergic rhinitis and eczema cases could have been misclassified since some topical corticosteroids and antihistamines are available as over-the-counter medications, meaning that lower specificity would be expected based on the algorithms.^39^ In addition, our results confirmed the previously reported within-individual association between each allergic condition with IBD^5,6^ and the familial aggregation for asthma hospitalizations and IBD.^19^ Familial co-aggregation results were seen across all IBD subtypes and allergic conditions with sufficient sample sizes, reflecting the frequent coexistence of asthma, allergic rhinitis, and eczema. The effect estimates were slightly larger for Crohn’s disease than for ulcerative colitis with allergic conditions, which was partially due to a higher familial aggregation in Crohn’s disease.^40,41^

Furthermore, our results concur with a large GWAS analysis carried out by Zhu et al^42^ in 2018 using UK Biobank data. Although their primary focus was on the shared genetic architecture between asthma and allergic diseases, they extended the analysis to include several other immune-related traits such as ulcerative colitis and Crohn’s disease. Interestingly, they found almost no evidence of genetic correlation.^42^ Based on our null results from newer GWAS summary statistics and PRS analysis, we infer that IBD and allergic disease as immune-related traits may be genetically distinct. However, we still could not completely rule out an unidentifiable weak or local genetic overlap at the locus or gene level, as it is possible that a small number of common variants with potential antagonistic pleiotropy may have annulled the effects of shared risk loci and led to an underestimated genetic correlation.^34^ Furthermore, the phenotype heterogeneity of allergic rhinitis GWASs based on relatively small sample size^43^ has challenged the performance of PRS, which may have also contributed to a dilution of genetic correlation in our analysis.

There is some contradiction in our results regarding the familial co-aggregation analyses indicating a shared familial component, which was not supported by the quantitative genetic modeling, indicating neither a shared genetic component nor a shared familial environment, but rather possibly a unique environmental component for IBD and eczema. Although we cannot completely rule out the possibility of a chance finding, we hypothesize that a possible unique environmental component explaining comorbidity could be that adult twin pairs may live in different geographic areas with different healthcare access or diagnostic practice that may influence how both eczema and IBD are diagnosed and/or treated. This discrepancy may also be due to the different sources of twin subcohorts vs population in the analysis, in particular concerning adult weight, lifestyle-related factors, and evolving treatments/diagnosis for eczema and IBD.

Furthermore, the weak bidirectional association between IBD and eczema observed in the PRS analyses indicates a possible shared genetic origin, in line with the familial co-aggregation results. This is consistent with a recent meta-analysis, supporting a bidirectional phenotypic association and suggesting several pleiotropic genes as possible candidates contributing to both diseases (eg, *STAT3*, *IL1RL1*, *IL18R1*).^44^ Furthermore, immunotherapy trials that use the same therapies targeting cytokine levels in inflammatory immune-mediated diseases including IBD and eczema provide further evidence for a common genetic pathway. For example, JAK inhibitors have been used as targeted therapies for both atopic dermatitis and IBD by suppressing the intracellular signaling via multiple cytokines involved in the pathological processes of these diseases.^45^

This study had a number of strengths. First, we were able to harness several different cohorts and a number of different population and molecular-based genetic methods to triangulate evidence. We have provided very robust evidence to support the lack of a shared genetic component between IBD and allergic diseases. Each of the methods had its individual strengths as well. The familial co-aggregation method was based on a very large population with long follow-up data that allowed in-depth interrogation of different levels of kinship data including for ulcerative colitis and Crohn’s disease. The ability to harness 5 different twin subcohorts from different time periods and different ages not only increases the power of the cross-twin cross-trait and quantitative modeling, but also improves the generalizability of the findings to a wider population. In addition, the cohort-based analyses were based on objective register-based diagnoses and medications rather than on subjective questionnaires, which strengthens the validity of the findings. Furthermore, we had access not only to phenotypic data for the twin cohorts, but also to genetic information, which allowed the generation and validation of PRSs. Finally, through recent publications of large GWASs of allergic diseases and IBD^43,46-48^ we were able to harness current genetic summary statistics for both the creation of reliable PRS and to perform LDSC regressions.

Our study also has some limitations which must be considered. First, both the IBD and allergic rhinitis GWASs were based on smaller populations compared with other diseases. Notably, for allergic rhinitis, the PRSs did not predict effectively despite a decent target sample size. Additionally, some extreme upper CI values were observed for the prediction of IBD using PRSs. Therefore, in the future, with larger GWASs for IBD and allergic rhinitis it would be advisable to revisit the PRS and LDSC analyses, especially between IBD and allergic rhinitis. Second, in the twin cohorts, we did not have enough power to separate out Crohn’s disease from ulcerative colitis reliably, which meant that we could not run the cross-twin cross-trait, quantitative modeling, and allergic disease PRS analyses separately for each IBD subtype, but rather treated IBD as a group. However, we were able to run the PRS in the other direction, using ulcerative colitis and Crohn’s disease and PRS and examining the associations with allergic phenotypes, revealing a possible association between Crohn’s disease and eczema. Larger studies are needed to investigate this further and illuminate whether a possible shared genetic architecture exists for Crohn’s disease and eczema. Another future line of study would be to assess the stability of genetic influence on the population variance of allergic diseases and IBD at different points in time, as this may affect shared genetic and environmental correlations at different ages.

## Conclusions

Utilizing population-wide, twin cohort, and GWAS data with several pedigree-based and molecular genetic based methods, we observed comorbidity between allergic diseases and IBD. However, our results did not provide robust evidence to support a shared genetic origin between IBD with asthma and allergic rhinitis. There was, notably, a weak signal that requires further investigation regarding shared genetic and unique environmental factors for the eczema and IBD comorbidity. Further research is needed to investigate the role of shared and/or unique environmental factors and the broader overlap between allergic diseases and gastrointestinal diseases in general.

## Supplementary data

Supplementary data is available at *Inflammatory Bowel Diseases* online.

izae027_suppl_Supplementary_Material
