# Supplementary material for: Comorbidity Between Inflammatory Bowel Disease and Asthma and Allergic Diseases: A Genetically Informed Study
Source: Inflamm Bowel Dis. 2024 Feb 27;30(9):1556–65. doi: 10.1093/ibd/izae027 (PMC11369071; doi:10.1093/ibd/izae027)
Supplement: izae027_suppl_Supplementary_Material [file izae027_suppl_supplementary_material.docx]

Supplementary materials

Comorbidity between inflammatory bowel disease and asthma and allergic diseases - a genetically informed study

Tong Gong, PhD^1^

Bronwyn K Brew, PhD^1,2^

Cecilia Lundholm, PhD^1^

Awad Smew, MD PhD^1^

Arvid Harder, MS^1^

Ralf Kuja-Halkola, PhD^1^

Jonas F. Ludvigsson, MD PhD^1,3^

Yi Lu, PhD^1^

Catarina Almqvist, MD PhD^1,4^

1. Department of Medical Epidemiology and Biostatistics, Karolinska Institute, Stockholm, Sweden.

2. Centre for Big Data Research in Health & School of Clinical Medicine, UNSW, Sydney, Australia.

3. School of Medical Sciences, Orebro Hospital, Orebro, Sweden.

4. Pediatric Allergy and Pulmonology Unit at Astrid Lindgren Children’s Hospital, Karolinska University Hospital, Stockholm, Sweden

Contents

[**Supplementary Methods** 3](#_Toc121730161)

[**Quality control, genotype imputation, and principal component analyses** 3](#_Toc121730162)

[**GWAS summary statistic data for identification of variants to include in the PRS estimation** 3](#_Toc121730163)

[**Bayesian summary statistics-based PRS method and PRS validation** 4](#_Toc121730164)

[**Table S1. Algorithms to identify cases of allergic diseases and inflammatory bowel diseases (IBD) from the national patient register (NPR) and the Swedish prescribed drug register (SPDR).** 5](#_Toc121730165)

[**Table S2. Detailed information of published GWAS summary data of allergic diseases and GERD used to calculate the polygenic risk scores and for the LD score regression analyses** 6](#_Toc121730166)

[**Table S3. Study characteristics of the probands and relatives in the proband-relative pairs.** 8](#_Toc121730167)

[**Table S4. Within-individual association and familial co-aggregation of allergic diseases and IBD (odds ratios and 95% confidence intervals)** 9](#_Toc121730168)

[**Table S5. Univariate (within-trait cross twin) and bivariate (phenotypic within individual, and cross-twin cross-trait) correlations for IBD and allergic diseases by sex for the five sub-cohorts of the Swedish Twin Register.** 11](#_Toc121730169)

[**Table S6. Quantitative genetic modelling - Bivariate Cholesky models. Genetic and environmental parameter estimates for Asthma and IBD using 38,723 pairs of twins.** 12](#_Toc121730170)

[**Table S7. Quantitative genetic modelling - Bivariate Cholesky models. Genetic and environmental parameter estimates for Allergic rhinitis and IBD using 38,723 pairs of twins.** 13](#_Toc121730171)

[**Table S8. Quantitative genetic modelling - Bivariate Cholesky models. Genetic and environmental parameter estimates for eczema and IBD using 18,876 pairs of twins.** 14](#_Toc121730172)

[**Table S9. Validation of the IBD/UC/CD based on polygenic risk scores in each twin sub-cohort dataset.** 15](#_Toc121730173)

[**Table S10. Associations (Odds ratios and 95% confidence intervals) between IBD/UC/CD PRS and allergic disease phenotypes among 48186 twins with genotype data.** 16](#_Toc121730174)

[**Table S11. Associations (Odds ratios and 95% confidence intervals) between allergic disease PRS and IBD phenotype for each twin sub-cohort.** 17](#_Toc121730175)

[**Table S12. Estimations of genetic correlation (r_g_) between allergic diseases and IBD subtypes using LD score regression analyses** 18](#_Toc121730176)

[**Figure S1. Prediction of the traits based on polygenic risk scores in deciles among each target (twin sub-cohort) set.** 19](#_Toc121730177)

# **Supplementary Methods**

## **Quality control, genotype imputation, and principal component analyses**

During the quality control (QC) procedures of the genotype data, we removed low-quality markers, e.g. with call rates<98%, that deviate grossly from the Hardy-Weinberg Equilibrium (p-value< 1e-6), large allele frequency differences from the 1000 Genome European reference samples, and low quality score, i.e. mean GenCall scores<0.5. In total, we removed xx (~2%) samples with sample calling rate <98%, unusual heterozygosity, possible sample contamination, sex violation, and non-European ancestral outliers. Furthermore, MZ twins’ genotype data were imputed from their paired genotyped twin.

Genotype imputation was performed using1000 Genome data (Phase 3 Version 5) / The Haplotype Reference Consortium (HRC) 1.1 as the reference panel. Phasing was performed using Shapeit2 on each chromosome, and imputation was performed using Minimac3 on 5Mb chromosomal chunks (with the window of 1Mb on either side). After imputation, ~47M markers were available, and over 7M common variants (MAF≥1%) have high imputation quality (imputation R^2^≥ 0.8).

## **GWAS summary statistic data for identification of variants to include in the PRS estimation**

We have searched from GWAS Catalog, Pubmed, MedRxiv, and UK Biobank’s website and found several available summary statistic data for allergic diseases and GERD. We used the analyzed association results for each SNP from the discovery sets by far with the largest sample sizes.

First, we found the largest genome-wide association study of asthma to date (153763 cases and 1647022 controls) via meta-analysis across 18 biobanks spanning multiple countries and ancestries. Specifically, the European ancestry based summary statistics for asthma from 14 biobanks, i.e. BioMe, BioVU, CCPM, DECODE, ESTBB, FinnGen, GS, HUNT, Lifelines, MGB, MGI, QSKIN, UCLA, UKBB were used as discovery samples to estimate the PRS^1^. However, most of the GWAS on asthma phenotypes are under-powered. For example, Nick Shrine and others have published one study on moderate-to-severe asthma using 5135 cases and 25675 controls from the UK^2^. The eosinophilic asthma phenotype was under-powered in the UK Biobank sample with 2302 cases and 358892 controls. No GWAS reported on allergic asthma. We could only use one powerful GWAS (based on UK Biobank sample) on childhood-onset asthma (COA) and adult-onset asthma (AOA) was available (COA cases: 13962, AOA cases: 26582, common set of controls: 300671)^3^.

Second, we have chosen the largest genome-wide meta-analysis published by far on hay fever/ allergic rhinitis (AR) (59762 cases and 152358 controls) to be our discovery sample^4^. Non-allergic rhinitis with 2028 cases and 9606 controls was not used due to small sample size. The authors combined data from children 6 years and above as well as adult participants. AR was defined as individuals having either a diagnosis or symptoms of AR depended on cohort-specific data availability (23andMe, UKBB, deCODE being the largest three studies together with 22 researcher-led cohorts).

Third, we have chosen the summary statistics data from the largest genome-wide meta analysis on eczema / atopic dermatitis using participants from the FinnGen, Estonian Biobank and the UK Biobank with European ancestry (22474 cases and 774187 controls)^5^. Phenotypic definition for eczema was based on diagnostic records with relevant ICD-codes (ICD-10 L20; ICD-9 6918; ICD-8: 691) among adults.

Fourth, we have chosen one of the largest genome-wide meta-analysis for inflammatory bowel disease (IBD), and the two subtypes ulcerative colitis (UC) and crohn’s disease (CD), which consists of 25042 IBD | 12366 UC | 12194 CD cases and 34915 | 33609 | 28072 controls. The UK HumanCoreExome genotyped IBD study has used diagnosis with endoscopic, histopathological and radiological criteria to define IBD cases. It was not stated in the manuscript or the supplementary information how IBD was defined in other cohorts which were previously analyzed^6^.

## **Bayesian summary statistics-based PRS method and PRS validation**

Regarding the process of validation, the PRS prediction accuracy and performance was assessed by using the Area Under the Receiver Operating Curve (AUC), odds ratios (OR) by decile (i.e. checking the sign of the regression coefficient in the expected direction), and Nagelkerke Pseudo-R^2^. We ran a series logistic regression models with clustered standard errors accounting for the twin relatedness and adjusting for the population stratification for each target set. We observed that PRS of asthma and IBD has a better prediction power than the allergic rhinitis and eczema (See Table S9 and Figure S1). For example, OR for phenotypic asthma in STAGE, TwinGene, and SALTY were increased accordingly by a higher percentile of PRS of asthma. The AUC values for the PRS of asthma were ranging from 0.60 to 0.62 by target sets.

# **Table S1. Algorithms to identify cases of allergic diseases and inflammatory bowel diseases (IBD) from the national patient register (NPR) and the Swedish prescribed drug register (SPDR).**

| **Disease** | **Algorithms to define cases during follow-up (NPR from birth to December 2020 and SPDR from July 2005 to July 2021)** |
| --- | --- |
| Asthma^7^ | 1. Having ≥ 1 asthma-relevant diagnosis (ICD-10 codes: J45-J46) from the NPR. 2. Or having ≥ 2 dispensed prescriptions of inhaled corticosteroids (ICS) (ATC-code: R03BA) and/or leukotriene antagonists (LTRA) (ATC-code: R03DC03) and/or combination of beta-2 agonists and ICS (ATC-codes: R03AK06, R03AK07) from SPDR independent on time between dispenses. 3. Or having ≥ 3 ICS and/or LTRA and/or combination and/or beta-2 agonists (ATC-codes: R03AC02, R03AC03, R03AC12, R03AC13) from SPDR within 12-month period.   Excluding COPD diagnoses |
| Allergic rhinitis^8^ | 1. Having ≥ 1 allergic rhinitis-relevant diagnosis (ICD-10 codes: J30, J31.0) from the NPR. 2. Or having having ≥ 2 dispensed prescriptions of ICS for rhinitis (ATC-code: R01AD) from the SPDR but no diagnoses of nasal polyps, acute sinusitis, or chronic sinusitis (ICD-10 codes: J33, J01, J32) from the NPR. 3. Or having ≥ 2 dispensed prescriptions of antihistamine for systemic use (ATC-code: R06A) from the SPDR but no diagnoses of pruritus or allergic urticarial (ICD-10 codes: L29, DL50) from the NPR. 4. Or having ≥ 1 dispensed prescription of allergen-specific immunotherapy (ATC-code: V01A) or prescription for allergic conjunctivitis (ATC-code: S01GX) from the SPDR. |
| Eczema^8^ | 1. Having ≥ 1 Atopic dermatitis-relevant diagnosis (ICD-10 codes: L20, L308C) from NPR. 2. Or having ≥ 1 dispensed prescription of tacrolimus, pimecrolimus (ATC-code: D11AH) from the SPDR without any of the exclusion critera specified in the Appendix A1 of Henriksen et al. 3. Or having ≥ 2 dispensed prescriptions of corticosteroids for topical use (ATC-code: D07) from the SPDR without any of the exclusion critera specified in the Appendix A1 of Henriksen et al. |
| Ulcerative colitis (UC)^9^ | Having ≥ 2 registered diagnostic records of UC from the NPR (ICD-8/9/10 codes: 563.10 or 569.02/556/K51) and no diagnostic records for the CD or NS-IBD. |
| Crohn’s disease (CD)^9^ | Having ≥ 2 registered diagnostic records of CD from the NPR (ICD-8/9/10 codes: 563.00/555/K50) and no diagnostic records for the UC or NS-IBD. |
| Non-specified IBD (NS-IBD) ^9^ | Having ≥ 2 NS-IBD diagnosis (ICD-8 codes:563.98/563.99) or having relevant diagnostic records of both UC and CD, meaning that the physicians failed to differentiate between UC or CD from the NPR. |
| IBD^9^ | Having ≥ 2 registered diagnoses of UC or CD or NS-IBD from the NPR. |

# **Table S2. Detailed information of published GWAS summary data of allergic diseases and GERD used to calculate the polygenic risk scores and for the LD score regression analyses**

| Source | PMID | Phenotype | Sample size | Reference panel used | Statistical method used | SNP-based heritability reported | Summary data download URL |
| --- | --- | --- | --- | --- | --- | --- | --- |
| **Asthma** | |  |  |  |  |  |  |
| Ferriera MA et al. | 30929738 | Childhood-onset (COA) and adulthood-onset asthma (AOA) | 447628 (however, the UKB and QSKIN data is based on 327253 and 314633 individuals) | 1000 Genome Project | Linear mixed model + Logistic regression + inverse-variance-weighted fixed-effects meta-analysis | h^2^_SNP_ (COA)=25.6%, h^2^_SNP_ (AOA) = 10.6% | <https://genepi.qimr.edu.au/staff/manuelF/gwas_results/CHILD_ONSET_ASTHMA.20180501.allchr.assoc.GC.gz>  <https://genepi.qimr.edu.au/staff/manuelF/gwas_results/ADULT1_ADULT2_ONSET_ASTHMA.20180716.allchr.assoc.GC.gz> |
| Zhou W et al. | medRxiv | Asthma | 153763 cases / 1647022 controls | 1000 Genome Project + Human Genome Diversity Project | SAIGE or REGENIE by cohort and inverse-variance weighted fixed effect model for meta-analyses | h^2^_SNP_ = 8.7% | <https://github.com/globalbiobank> |
| **Allergic rhinitis** | | | | | | | |
| Waage J et al. | 30013184 | Allergic rhinitis (symptoms/ diagnosis) ever | 59762 cases/ 152358 controls. However, the downloaded summary data included 38838 individuals | UK10K | Inverse-variance weighted fixed effect model for meta-analyses | h^2^_SNP_ = 7.8% | https://hmgubox.helmholtz-muenchen.de/d/b55da086360c40118ae8/files/?p=/2018-05-11_EAGLE_AR.txt.gz |
| **Eczema** | | | | | | | |
| Sliz E et al. | 34454985 | Diagnosis of atopic dermatitis | 22474 cases / 774187 controls | Finnish population-specific SISu v3 + Estonian-specific reference panel + 1000 Genomes phase 3 | Inverse-variance weighted fixed effect model for meta-analyses | h^2^_SNP_ = 5.4% | <http://ftp.ebi.ac.uk/pub/databases/gwas/summary_statistics/GCST90027001-GCST90028000/GCST90027161/harmonised/34454985-GCST90027161-EFO_0000274.h.tsv.gz> |
| **IBD** | | | | | | | |
| De Lange K et al. | 28067908 | IBD diagnosed using accepted endoscopic, histopathological and radiological criteria, | 25042 cases / 34915 controls | 1000 Genomes phase 3 | Fixed effect model for meta-analyses | h^2^_SNP_ not directly reported | https://figshare.com/articles/dataset/GERD_GWAS_summary/8986589 |

# **Table S3. Study characteristics of the probands and relatives in the proband-relative pairs.**

|  |  | **Proband characteristics (n,%)**  **N=2,873,445** | **Characteristics or relatives in the proband-relative pairs (n,%)** | | | |
| --- | --- | --- | --- | --- | --- | --- |
|  |  |  | **Full-siblings**  **N= 3,293,364** | **Parents**  **N= 5,721,581** | **Maternal halfsiblings N= 524,926** | **Paternal halfsiblings N= 591,476** |
| Sex | male | 1,476,235 (51.4) | 1696302 (51.5) | 2848137 (49.8) | 269295 (51.3) | 301661 (51.0) |
|  | female | 1,397,210 (48.6) | 1597062 (48.5) | 2873444 (50.2) | 255631 (48.7) | 289815 (49.0) |
| Parity | first | 1302586 (45.3) | 1162004 (35.3) | - | 161681 (30.8) | 317046 (53.6) |
|  | second | 1036365 (36.1) | 1220969 (37.1) | - | 143369 (27.3) | 174938 (29.6) |
|  | third | 388812 (13.5) | 595076 (18.1) | - | 123187 (23.5) | 65247 (11.0) |
|  | fourth or later | 145682 (5.1) | 315315 (9.6) | - | 96689 (18.4) | 34245 (5.8) |
| IBD | | 20140 (0.7) | 22919(0.7) | 59560(1.0) | 3542(0.7) | 4126(0.7) |
| Ulcerative colitis | | 9666 (0.3) | 10934(0.3) | 32379(0.6) | 1637(0.3) | 1919(0.3) |
| Crohn’s disease | | 7528 (0.3) | 8651(0.3) | 18077(0.3) | 1398(0.3) | 1646(0.3) |
| Asthma | | 516346 (18.0) | 577160(17.5) | 767695(13.4) | 107677(20.5) | 116698(19.7) |
| Allergic rhinitis | | 878177 (30.6) | 945492(28.7) | 1929877(33.7) | 169603(32.3) | 191702(32.4) |
| Eczema | | 467284 (16.3) | 512964(15.6) | 984295(17.2) | 82055(15.6) | 96174(16.3) |
| Year of birth | mean (SD) | 2000.4 (8.3) | 2000.3 (7.7) | 1969.0 (9.4) | 1999.9 (7.6) | 2000.1 (7.9) |

# **Table S4. Within-individual association and familial co-aggregation of allergic diseases and IBD (odds ratios and 95% confidence intervals)**

|  |  | OR (95% CI) | | | | | | | | |
| --- | --- | --- | --- | --- | --- | --- | --- | --- | --- | --- |
|  |  | Asthma | | | Allergic rhinitis | | | Eczema | | |
| **Proband-relative categories** | by | Model 1 | Model 2 | Model 3 | Model 1 | Model 2 | Model 3 | Model 1 | Model 2 | Model 3 |
| Proband – within individual (n=2,873,445) | IBD | 1.19 (1.15, 1.24) | 1.35  (1.30, 1.40) | -  (-, -) | 1.61 (1.56, 1.65) | 1.43  (1.39, 1.48) | -  (-, -) | 1.40 (1.35, 1.46) | 1.59  (1.53, 1.65) | -  (-, -) |
|  | UC | 1.11 (1.05, 1.17) | 1.27  (1.20, 1.34) | -  (-, -) | 1.41 (1.35, 1.47) | 1.25  (1.20, 1.31) | -  (-, -) | 1.20 (1.13, 1.27) | 1.37  (1.29, 1.45) | -  (-, -) |
|  | CD | 1.33 (1.25, 1.41) | 1.47  (1.38, 1.56) | -  (-, -) | 1.83 (1.74, 1.92) | 1.64  (1.56, 1.72) | -  (-, -) | 1.63 (1.54, 1.73) | 1.80  (1.70, 1.91) | -  (-, -) |
|  |  |  |  |  |  |  |  |  |  |  |
| Monozygotic twins (13,028 pairs) * | IBD | 1.69  (1.03, 2.88) | 1.73  (1.05, 2.83) | 1.84  (1.11, 3.06) | 1.38  (0.87, 2.20) | 1.37  (0.86, 2.18) | 1.28  (0.79, 2.09) | 1.46  (0.82, 2.62) | - | 1.27  (0.70, 2.31) |
|  | UC | 1.36  (0.72, 2.54) | 1.39  (0.74, 2.59) | 1.45  (0.77, 2.75) | 0.97  (0.53, 1.78) | 0.95  (0.52, 1.74) | 0.83  (0.44, 1.57) | 1.35  (0.65, 2.79) | 1.26  (0.60. 2.63) | 1.29  (0.63, 2.63) |
|  | CD | 2.76  (1.32, 5.76) | 2.81  (1.35, 5.85) | 3.20  (1.46, 7.05) | 3.00  (1.52, 5.90) | 3.06  (1.55, 6.04) | 3.32  (1.59, 6.92) | 2.52  (1.14, 5.55) | 2.45  (1.11, 5.44) | 1.98  (0.79, 5.00) |
|  |  |  |  |  |  |  |  |  |  |  |
| Dizygotic twins (25,695 pairs) * | IBD | 0.68  (0.43, 1.07) | 0.73  (0.46, 1.15) | 0.69  (0.44, 1.10) | 1.22  (0.87, 1.74) | 1.22  (0.86, 1.73) | 1.16  (0.82, 1.65) | 1.16  (0.75, 1.79) | 1.03  (0.67, 1.59) | 0.98  (0.63, 1.52) |
|  | UC | 0.69  (0.40, 1.20) | 0.75  (0.43, 1.31) | 0.69  (0.40, 1.22) | 1.21  (0.79, 1.86) | 1.20  (0.78, 1.84) | 1.13  (0.73, 1.73) | 1.14  (0.67, 1.95) | 1.00  (0.58, 1.71) | 0.96  (0.56, 1.66) |
|  | CD | 0.68  (0.34, 1.36) | 0.71  (0.36, 1.42) | 0.69  (0.34, 1.39) | 1.19  (0.70, 2.02) | 1.19  (0.69, 2.03) | 1.13  (0.66, 1.95) | 0.83  (0.39, 1.77) | 0.77  (0.36, 1.65) | 0.72  (0.33, 1.57) |
|  |  |  |  |  |  |  |  |  |  |  |
| Proband & Full-siblings | IBD | 1.05 (1.00, 1.09) | 1.16  (1.12, 1.21) | 1.15  (1.11, 1.20) | 1.30 (1.26, 1.34) | 1.19  (1.15, 1.23) | 1.17  (1.14, 1.21) | 1.07 (1.02, 1.11) | 1.16  (1.12, 1.22) | 1.14  (1.09, 1.19) |
|  | UC | 0.98 (0.92, 1.04) | 1.10  (1.04, 1.17) | 1.10  (1.03, 1.16) | 1.20 (1.15, 1.26) | 1.09  (1.04, 1.15) | 1.09  (1.04, 1.14) | 1.01 (0.95, 1.07) | 1.11  (1.04, 1.18) | 1.10  (1.03, 1.17) |
|  | CD | 1.16 (1.09, 1.23) | 1.26  (1.18, 1.34) | 1.25  (1.17, 1.33) | 1.40 (1.33, 1.47) | 1.29  (1.23, 1.36) | 1.28  (1.21, 1.34) | 1.14 (1.07, 1.22) | 1.22  (1.14, 1.31) | 1.20  (1.12, 1.29) |
|  |  |  |  |  |  |  |  |  |  |  |
| Proband & Parents | IBD | 1.14 (1.12, 1.16) | 1.15  (1.13, 1.17) | 1.14  (1.12, 1.16) | 1.16 (1.14, 1.18) | 1.15  (1.13, 1.17) | 1.14  (1.13, 1.16) | 1.10 (1.07, 1.12) | 1.10  (1.08, 1.13) | 1.09  (1.07, 1.12) |
|  | UC | 1.06 (1.04, 1.09) | 1.08  (1.05, 1.10) | 1.07  (1.05, 1.10) | 1.10  (1.08, 1.13) | 1.09  (1.07, 1.12) | 1.09 (1.07, 1.11) | 1.05 (1.02, 1.08) | 1.05  (1.02, 1.08) | 1.05  (1.02, 1.08) |
|  | CD | 1.24 (1.20, 1.29) | 1.25  (1.21, 1.29) | 1.25  (1.20, 1.29) | 1.25 (1.22, 1.29) | 1.25  (1.21, 1.28) | 1.24  (1.20, 1.27) | 1.16 (1.11, 1.20) | 1.17  (1.12, 1.21) | 1.16  (1.12, 1.20) |
|  |  |  |  |  |  |  |  |  |  |  |
| Proband & Maternal half-siblings | IBD | 1.04 (0.94, 1.15) | 1.06  (0.96, 1.18) | 1.06  (0.96, 1.18) | 1.12 (1.03, 1.21) | 1.10  (1.01, 1.19) | 1.10  (1.01, 1.19) | 1.01 (0.91, 1.11) | 1.03  (0.93, 1.15) | 1.03  (0.93, 1.15) |
|  | UC | 1.09 (0.94, 1.25) | 1.10  (0.96, 1.27) | 1.10  (0.96, 1.27) | 1.07 (0.96, 1.20) | 1.05  (0.94, 1.18) | 1.05  (0.94, 1.18) | 0.96 (0.83, 1.12) | 1.00  (0.86, 1.16) | 1.00  (0.86, 1.16) |
|  | CD | 0.95 (0.81, 1.12) | 0.97  (0.82, 1.14) | 0.97  (0.82, 1.14) | 1.16 (1.02, 1.31) | 1.14  (1.01, 1.30) | 1.14  (1.01, 1.30) | 1.04 (0.88, 1.22) | 1.06 (0.90, 1.25) | 1.06 (0.90, 1.25) |
|  |  |  |  |  |  |  |  |  |  |  |
| Proband & Paternal half-siblings | IBD | 1.00 (0.92, 1.09) | 1.00 (0.92, 1.09) | 1.00 (0.92, 1.09) | 1.02 (0.95, 1.09) | 1.04  (0.97, 1.12) | 1.04  (0.97, 1.12) | 1.05 (0.96, 1.14) | 1.05 (0.96, 1.14) | 1.05 (0.96, 1.14) |
|  | UC | 0.98 (0.87, 1.10) | 0.98 (0.87, 1.10) | 0.98 (0.87, 1.10) | 1.01 (0.91, 1.11) | 1.02  (0.93, 1.13) | 1.02  (0.93, 1.13) | 1.07 (0.94, 1.22) | 1.07 (0.94, 1.22) | 1.07 (0.94, 1.22) |
|  | CD | 1.00 (0.88, 1.14) | 1.01  (0.88, 1.15) | 1.01  (0.88, 1.15) | 1.02 (0.92, 1.13) | 1.05  (0.94, 1.17) | 1.05  (0.94, 1.17) | 0.98 (0.86, 1.12) | 0.98 (0.85, 1.12) | 0.98 (0.85, 1.12) |
|  |  |  |  |  |  |  |  |  |  |  |

*The MZ and DZ twin analyses were based on the sub-cohorts of twins born 1911-2012 identified from the Swedish Twin Registry, in order to maximize the statistical power. See the methods section on the sub-cohort description.

Model 1 with no adjustment.

Model 2 adjusted for birth year, sex, and parity

Model 3 adjusted for birth year, sex, parity, and relative's IBD/UC/CD.

# **Table S5. Univariate (within-trait cross twin) and bivariate (phenotypic within individual, and cross-twin cross-trait) correlations for IBD and allergic diseases by sex for the five sub-cohorts of the Swedish Twin Register.**

|  | **Univariate within-trait cross-twin Intraclass correlations** | | | | **Bivariate phenotypic and cross-twin cross-trait (CTCT) correlations** | | | | | |
| --- | --- | --- | --- | --- | --- | --- | --- | --- | --- | --- |
|  |  |  |  |  | **Asthma & IBD** | | **Allergic Rhinitis & IBD** | | **Eczema & IBD** | |
| **Type of relatives** | **IBD** | **Asthma** | **Allergic Rhinitis** | **Eczema** | **Phenotypic**  **correlation** | **CTCT**  **correlation** | **Phenotypic**  **correlation** | **CTCT**  **correlation** | **Phenotypic**  **correlation** | **CTCT correlation** |
| **MZ twins (n=13,028 pairs)** |  |  |  |  |  |  |  |  |  |  |
| Females | 0.78  (0.71, 0.85) | 0.75  (0.73, 0.77) | 0.54 (0.51, 0.57) | 0.47 (0.43, 0.51) | -0.01 (-0.13, 0.12) | 0.11 (0.00, 0.22) | 0.07 (-0.04, 0.18) | 0.05 (-0.05, 0.16) | 0.13 (0.01, 0.24) | 0.07 (-0.05, 0.19) |
| Males | 0.64  (0.55, 0.74) | 0.67  (0.65, 0.70) | 0.51 (0.48, 0.53) | 0.40 (0.37, 0.44) | 0.07 (-0.03, 0.18) | 0.01 (-0.10, 0.12) | 0.10 (0.00, 0.19) | 0.03 (-0.07, 0.13) | 0.10 (0.00, 0.20) | 0.10 (0.00, 0.20) |
| **DZ twins (n=25,695 pairs)** |  |  |  |  |  |  |  |  |  |  |
| Females | 0.57  (0.45, 0.68) | 0.45  (0.42, 0.49) | 0.32 (0.29, 0.36) | 0.27 (0.23, 0.32) | 0.13 (0.03, 0.23) | -0.09 (-0.21, 0.02) | 0.05 (-0.05, 0.15) | -0.04 (-0.14, 0.07) | 0.06 (-0.05, 0.17) | -0.16 (-0.30, -0.02) |
| Males | 0.53  (0.43, 0.64) | 0.47  (0.44, 0.50) | 0.33 (0.30, 0.36) | 0.26 (0.22, 0.29) | 0.00 (-0.10, 0.09) | -0.08 (-0.18, 0.03) | 0.12 (0.04, 0.21) | 0.00 (-0.09, 0.09) | 0.10 (0.01, 0.19) | 0.03 (-0.07, 0.13) |
| Opposite sex | 0.30  (0.18, 0.42) | 0.29  (0.26, 0.32) | 0.22 (0.19, 0.24) | 0.18 (0.15, 0.21) | 0.04 (-0.04, 0.12) | -0.03 (-0.12, 0.05) | 0.04 (-0.03, 0.11) | 0.04 (-0.03, 0.12) | 0.14 (0.06, 0.21) | -0.03 (-0.12, 0.05) |

# **Table S6. Quantitative genetic modelling - Bivariate Cholesky models. Genetic and environmental parameter estimates for Asthma and IBD using 38,723 pairs of twins.**

|  | Models | | |
| --- | --- | --- | --- |
| Parameters | ACE | ADE | AE |
| *Asthma, % (95% CI)* |  |  |  |
| Asthma due to A | 0.65 (0.59, 0.71) | 0.72 (0.70, 0.74) | 0.72 (0.70, 0.74) |
| Asthma due to D | Na | 0.00 (0.00, 0.00) | Na |
| Asthma due to H | Na | 0.72 (0.70, 0.74) | Na |
| Asthma due to C | 0.06 (0.01, 0.11) | Na | Na |
| Asthma due to E | 0.29 (0.27, 0.31) | 0.28 (0.26, 0.30) | 0.28 (0.26, 0.30) |
| *IBD, % (95% CI)* |  |  |  |
| IBD due to A | 0.52 (0.31, 0.73) | 0.74 (0.67, 0.81) | 0.74 (0.67, 0.81) |
| IBD due to D | Na | 0.00 (0.00, 0.00) | Na |
| IBD due to H | Na | 0.74 (0.67, 0.81) | Na |
| IBD due to C | 0.20 (0.03, 0.36) | Na | Na |
| IBD due to E | 0.29 (0.20, 0.37) | 0.26 (0.19, 0.33) | 0.26 (0.19, 0.33) |
| *Bivariate explained covariance, % (95% CI)* |  |  |  |
| Covariance due to A | 2.94 (0.58, 5.03) | 0.61 (-0.23, 1.46) | 0.61 (-0.23, 1.46) |
| Covariance due to D | Na | 0.00 (0.00, 0.00) | Na |
| Covariance due to H | Na | 0.61 (-0.23, 1.46) | Na |
| Covariance due to C | -1.89 (-3.67, -0.11) | Na | Na |
| Covariance due to E | -0.05 (-1.06, 0.96) | 0.39 (-0.46, 1.23) | 0.39 (-0.46, 1.23) |
| *Bivariate correlations, % (95% CI)* |  |  |  |
| rA | 0.30 (0.09, 0.51) | 0.05 (-0.03, 0.12) | 0.05 (-0.03, 0.12) |
| rD | Na | 0.97 (-4489.90, 4491.85) | Na |
| rH | Na | 0.05 (-0.38, 0.47) | Na |
| rC | -1.00 (-1.00, -1.00) | Na | Na |
| rE | -0.01 (-0.21, 0.19) | 0.08 (-0.10, 0.27) | 0.08 (-0.10, 0.27) |
| *Model fitting* | | | |
| Akaike Information Criterion | 31764.26 | 31767.84 | 31761.84 |
| p-value from likelihood ratio test |  |  |  |
| ACE VS AE | 0.310 | | |
| ADE VS AE | 1 | | |

All models were adjusted for sex and birth year (continuous, standardized).

Abbreviations: A, additive genetic component; D, non-additive/ dominant genetic component; H, broad-sense heritability component, which is A+D; C, shared environmental component; E, non-shared environmental component (including measurement errors); rA, additive genetic correlation; rD dominant genetic correlation; rH, correlation atrributable to broad-sense heritability; rC, shared environmental correlation; rE, non-shared environmental correlation; na, not applicable.

# **Table S7. Quantitative genetic modelling - Bivariate Cholesky models. Genetic and environmental parameter estimates for Allergic rhinitis and IBD using 38,723 pairs of twins.**

|  | Models | | |
| --- | --- | --- | --- |
| Parameters | ACE | ADE | AE |
| *Allergic rhinitis, % (95% CI)* |  |  |  |
| Allergic rhinitis due to A | 0.49 (0.41, 0.56) | 0.53 (0.51, 0.55) | 0.53 (0.51, 0.55) |
| Allergic rhinitis due to D | Na | 0.00 (0.00, 0.00) | Na |
| Allergic rhinitis due to H | Na | 0.53 (0.51, 0.55) | Na |
| Allergic rhinitis due to C | 0.03 (-0.02, 0.09) | Na | Na |
| Allergic rhinitis due to E | 0.48 (0.45, 0.51) | 0.47 (0.45, 0.49) | 0.47 (0.45, 0.49) |
| *IBD, % (95% CI)* |  |  |  |
| IBD trait due to A | 0.59 (0.01, 1.17) | 0.74 (0.67, 0.82) | 0.74 (0.67, 0.82) |
| IBD trait due to D | Na | 0.00 (0.00, 0.00) | na |
| IBD trait due to H | Na | 0.74 (0.67, 0.82) | na |
| IBD trait due to C | 0.13 (-0.33, 0.60) | Na | na |
| IBD trait due to E | 0.28 (0.15, 0.40) | 0.26 (0.18, 0.33) | 0.26 (0.18, 0.33) |
| *Bivariate explained covariance, % (95% CI)* |  |  |  |
| Covariance due to A | 0.44 (-1.58, 2.46) | 0.47 (-0.21, 1.14) | 0.47 (-0.21, 1.14) |
| Covariance due to D | Na | 0.00 (0.00, 0.00) | Na |
| Covariance due to H | Na | 0.47 (-0.21, 1.14) | Na |
| Covariance due to C | 0.02 (-1.44, 1.49) | Na | Na |
| Covariance due to E | 0.54 (-0.27, 1.34) | 0.53 (-0.14, 1.21) | 0.53 (-0.14, 1.21) |
| *Bivariate correlations, % (95% CI)* |  |  |  |
| rA | 0.07 (-0.23, 0.37) | 0.06 (-0.03, 0.15) | 0.06 (-0.03, 0.15) |
| rD | Na | -0.11 (-6080.69, 6080.91) | Na |
| rH | Na | 0.06 (-0.35, 0.47) | Na |
| rC | 0.03 (-1.67, 1.72) | Na | Na |
| rE | 0.12 (-0.07, 0.30) | 0.12 (-0.04, 0.29) | 0.12 (-0.04, 0.29) |
| *Model fitting* | | | |
| Akaike Information Criterion | 40890.41 | 40892.52 | 40886.41 |
| p-value from likelihood ratio test |  |  |  |
| ACE VS AE | 0.572 | | |
| ADE VS AE | 1 | | |

All models were adjusted for sex and birth year (continuous, standardized).

Abbreviations: A, additive genetic component; D, non-additive/ dominant genetic component; H, broad-sense heritability component, which is A+D; C, shared environmental component; E, non-shared environmental component (including measurement errors); rA, additive genetic correlation; rD dominant genetic correlation; rH, correlation atrributable to broad-sense heritability; rC, shared environmental correlation; rE, non-shared environmental correlation; na, not applicable.

# **Table S8. Quantitative genetic modelling - Bivariate Cholesky models. Genetic and environmental parameter estimates for eczema and IBD using 18,876 pairs of twins.**

|  | Models | | |
| --- | --- | --- | --- |
| Parameters | ACE | ADE | AE |
| *Eczema, % (95% CI)* |  |  |  |
| Eczema due to A | 0.39 (0.32, 0.47) | 0.41 (0.30, 0.51) | 0.44 (0.41, 0.47) |
| Eczema due to D | Na | 0.03 (-0.08, 0.15) | Na |
| Eczema due to H | Na | 0.56 (0.52, 0.59) | Na |
| Eczema due to C | 0.04 (-0.02, 0.09) | Na | Na |
| Eczema due to E | 0.57 (0.54, 0.61) | 0.44 (0.41, 0.48) | 0.56 (0.53, 0.59) |
| *IBD, % (95% CI)* |  |  |  |
| *IBD* due to A | 0.52 (na, na) | 0.70 (0.48, 0.92) | 0.74 (0.67, 0.82) |
| *IBD* due to D | Na | 0.05 (-0.17, 0.27) | Na |
| *IBD* due to H | Na | 0.75 (0.67, 0.82) | Na |
| *IBD* due to C | 0.20 (na, na) | Na | Na |
| *IBD* due to E | 0.29 (0.23, 0.35) | 0.25 (0.18, 0.33) | 0.26 (0.18, 0.33) |
| *Bivariate explained covariance, % (95% CI)* |  |  |  |
| Covariance due to A | 1.46 (0.60, 2.32) | 0.00 (-1.44, 1.43) | 0.36 (-0.26, 0.99) |
| Covariance due to D | Na | 0.43 (-1.07, 1.92) | Na |
| Covariance due to H | Na | 0.42 (-0.23, 1.07) | Na |
| Covariance due to C | -0.86 (-1.44, -0.29) | na | Na |
| Covariance due to E | 0.40 (-0.35, 1.15) | 0.58 (-0.07, 1.23) | 0.64 (0.01, 1.26) |
| *Bivariate correlations, % (95% CI)* |  |  |  |
| rA | 0.32 (0.18, 0.45) | 0.00 (-0.26, 0.26) | 0.06 (-0.05, 0.17) |
| rD | Na | 1.00 (1.00, 1.00) | Na |
| rH | Na | 0.28 (-0.06, 0.62) | Na |
| rC | -1.00 (-1.00, -1.00) | Na | Na |
| rE | 0.10 (-0.08, 0.28) | 0.15 (-0.03, 0.32) | 0.16 (0.00, 0.32) |
| *Model fitting* | | | |
| Akaike Information Criterion | 33228.58 | 33234.82 | 33228.28 |
| p-value from likelihood ratio test |  |  |  |
| ACE VS AE | 0.096 | | |
| ADE VS AE | 0.992 | | |

All models were adjusted for sex and birth year (continuous, standardized).

Abbreviations: A, additive genetic component; D, non-additive/ dominant genetic component; H, broad-sense heritability component, which is A+D; C, shared environmental component; E, non-shared environmental component (including measurement errors); rA, additive genetic correlation; rD dominant genetic correlation; rH, correlation atrributable to broad-sense heritability; rC, shared environmental correlation; rE, non-shared environmental correlation; na, not applicable.

# **Table S9. Validation of the IBD/UC/CD based on polygenic risk scores in each twin sub-cohort dataset.**

| Cohort | Phenotype | n_Case_: n_control_ | Nagelkerke R^2^ | AUC |
| --- | --- | --- | --- | --- |
| CATSS wave 1 | Asthma | 2384:10816 | 0.034 | 0.6032 |
|  | Allergic rhinitis | 3061:10139 | 0.002 | 0.5268 |
|  | Eczema | 1657:11543 | 0.023 | 0.5900 |
|  | IBD | 46:13154 | 0.073 | 0.7225 |
|  | UC | 27:13173 | 0.078 | 0.7755 |
|  | CD | 26:13174 | 0.070 | 0.7415 |
| CATSS wave 2 | Asthma | 925:3894 | 0.036 | 0.6056 |
|  | Allergic rhinitis | 964:3855 | 0.002 | 0.5232 |
|  | Eczema | 627:4192 | 0.018 | 0.5833 |
|  | IBD | 7:4812 | 0.164 | 0.8836 |
|  | UC | 0:4819 | - | - |
|  | CD | 7:4812 | 0.146 | 0.8601 |
| YATSS | Asthma | 379:2893 | 0.058 | 0.6447 |
|  | Allergic rhinitis | 829:2443 | 0.008 | 0.5350 |
|  | Eczema | 356:3239 | 0.022 | 0.5934 |
|  | IBD | 33:3239 | 0.071 | 0.7076 |
|  | UC | 24:3248 | 0.094 | 0.7458 |
|  | CD | 10:3262 | 0.024 | 0.6663 |
| STAGE | Asthma | 977:8612 | 0.057 | 0.6522 |
|  | Allergic rhinitis | 2501:7088 | 0.001 | 0.5117 |
|  | Eczema | 1015:8574 | 0.006 | 0.5458 |
|  | IBD | 115:9474 | 0.064 | 0.7185 |
|  | UC | 78:9511 | 0.066 | 0.7335 |
|  | CD | 46:9543 | 0.066 | 0.7349 |
| TwinGene | Asthma | 1449:9459 | 0.026 | 0.5975 |
|  | Allergic rhinitis | 2482:8426 | 0.001 | 0.5148 |
|  | Eczema | 1800:9108 | 0.003 | 0.5269 |
|  | IBD | 139:10769 | 0.043 | 0.6750 |
|  | UC | 100:10808 | 0.024 | 0.6247 |
|  | CD | 44:10864 | 0.061 | 0.7370 |
| SALTY | Asthma | 841:5557 | 0.041 | 0.6255 |
|  | Allergic rhinitis | 1547:4851 | 0.001 | 0.5174 |
|  | Eczema | 895:5503 | 0.003 | 0.5385 |
|  | IBD | 91:6307 | 0.064 | 0.7206 |
|  | UC | 61:6337 | 0.035 | 0.6718 |
|  | CD | 37:6361 | 0.104 | 0.8068 |

# **Table S10. Associations (Odds ratios and 95% confidence intervals) between IBD/UC/CD PRS and allergic disease phenotypes among 48186 twins with genotype data.**

| PRS | Phenotype | Number of cases | Overall OR (95% CI) | |
| --- | --- | --- | --- | --- |
|  |  |  | Model 1 | Model 2 |
| IBD | Asthma | 6955 | 1.02 (0.99, 1.04) | 1.02 (0.99, 1.05) |
| UC | Asthma | 6955 | 0.98 (0.96, 1.01) | 0.98 (0.96, 1.01) |
| CD | Asthma | 6955 | 1.02 (1.00, 1.05) | 1.03 (1.00, 1.06) |
| IBD | Allergic rhinitis | 11384 | 1.03 (1.01, 1.05) | 1.02 (1.00, 1.05) |
| UC | Allergic rhinitis | 11384 | 1.00 (0.98, 1.03) | 1.00 (0.98, 1.02) |
| CD | Allergic rhinitis | 11384 | 1.04 (1.01, 1.06) | 1.04 (1.01, 1.06) |
| IBD | Eczema | 6350 | 1.09 (1.06, 1.12) | 1.09 (1.06, 1.12) |
| UC | Eczema | 6350 | 1.04 (1.02, 1.07) | 1.04 (1.01, 1.07) |
| CD | Eczema | 6350 | 1.09 (1.06, 1.12) | 1.09 (1.06, 1.12) |

Model 1 with no adjustment.

Model 2 adjusted for birth year, sex, and interaction term between top 5 principal components and cohort (i.e. CATSS wave 1, CATSS wave 2, YATSS, STAGE, TwinGene, SALTY).

# **Table S11. Associations (Odds ratios and 95% confidence intervals) between allergic disease PRS and IBD phenotype for each twin sub-cohort.**

| PRS | Phenotype | Number of IBD cases* | Overall OR (95% CI) | |
| --- | --- | --- | --- | --- |
|  |  |  | Model 1 | Model 2 |
| Asthma | IBD | 431 | 1.02 (0.93, 1.12) | 1.02 (0.93, 1.12) |
| COA | IBD | 431 | 0.98 (0.89, 1.07) | 0.98 (0.88, 1.07) |
| AOA | IBD | 431 | 1.04 (0.94, 1.14) | 1.02 (0.92, 1.13) |
| Allergic rhinitis | IBD | 431 | 1.03 (0.93, 1.14) | 1.03 (0.93, 1.14) |
| Eczema | IBD | 431 | 1.15 (1.05, 1.26) | 1.15 (1.05, 1.27) |
| Asthma | UC | 290 | 0.98 (0.87, 1.10) | 0.99 (0.88, 1.11) |
| COA | UC | 290 | 0.96 (0.86, 1.08) | 0.97 (0.86, 1.09) |
| AOA | UC | 290 | 0.99 (0.89, 1.12) | 0.98 (0.87, 1.11) |
| Allergic rhinitis | UC | 290 | 1.03 (0.91, 1.16) | 1.02 (0.91, 1.16) |
| Eczema | UC | 290 | 1.15 (1.02, 1.29) | 1.15 (1.03, 1.29) |
| Asthma | CD | 170 | 1.04 (0.89, 1.20) | 1.03 (0.88, 1.20) |
| COA | CD | 170 | 1.05 (0.90, 1.22) | 1.03 (0.88, 1.21) |
| AOA | CD | 170 | 1.10 (0.94, 1.29) | 1.08 (0.91, 1.27) |
| Allergic rhinitis | CD | 170 | 1.05 (0.89, 1.24) | 1.06 (0.89, 1.25) |
| Eczema | CD | 170 | 1.17 (1.00, 1.36) | 1.18 (1.01, 1.39) |

Abbreviation: AOA-adulthood onset asthma, COA-childhood onset asthma

Model 1 with no adjustment.

Model 2 adjusted for birth year, sex and interaction term between top 5 principal components and cohort (i.e. CATSS wave 1, CATSS wave 2, YATSS, STAGE, TwinGene, SALTY).

# **Table S12. Estimations of genetic correlation (r_g_) between allergic diseases and IBD subtypes using LD score regression analyses**

| **IBD and subtypes** | **Allergic diseases** | **N for allergic disease**  **(sample prevalence %,**  **population prevalence %)** | **h^2^ _SNP (allergic disease):_ h^2^ _SNP (IBD)_** | **r_g_ (SE) of allergic diseases with IBD^1^** | **p-value^3^** |
| --- | --- | --- | --- | --- | --- |
| UC | Asthma | 1800785 (8.5%, 8%) | 0.0826: 0.1417 | -0.0622 (0.0415) | 0.1343 |
| UC | Adulthood onset asthma | 327253 (8.1%, 8%) | 0.1297:0.1423 | -0.0283 (0.0454) | 0.5338 |
| UC | Childhood onset asthma | 314633 (4.4%, 5%) | 0.3321: 0.1423 | -0.006 (0.054) | 0.911 |
| UC | Allergic rhinitis | 38838 (27.2%, 25%)^2^ | 0.1201:0.1439 | 0.0196 (0.0811) | 0.8011 |
| UC | Eczema | 796661 (2.8%, 3%) | 0.0829:0.1434 | -0.0043 (0.022) | 0.8442 |
| CD | Asthma | 1800785 (8.5%, 8%) | 0.0827: 0.2507 | 0.114 (0.0444) | 0.0102 |
| CD | Adulthood onset asthma | 327253 (5.9%, 5%) | 0.1298: 0.2486 | 0.0834 (0.0514) | 0.1042 |
| CD | Childhood onset asthma | 314633 (3.1%, 10%) | 0.3324: 0.2485 | 0.0326 (0.0511) | 0.5236 |
| CD | Allergic rhinitis | 38838 (27.2%, 25%)^2^ | 0.1199:0.2508 | 0.0923 (0.0689) | 0.1801 |
| CD | Eczema | 796661 (2.8%, 3%) | 0.083:0.2494 | 0.0146 (0.0186) | 0.4337 |

Note:

1. We downloaded the summary data of IBD/UC/CD from de Lange K et al, involving 59957/45975/40266 European-ancestry individuals (reported sample prevalence at 41.8%, and assume the population prevalence at 1% for IBD; 26.9% and 0.5% for UC and 30.3% and 0.5% for CD).

2. The GWAS of allergic rhinitis conducted by Wagge J et al involved 120482 cases and 770,885 controls of European ancestry. However, the actual sample size from the downloadable summary data was based on 38838 individuals.

3. Bonferroni corrected significance across 10 tested LDSC for genetic correlation should be at 0.005.

# **Figure S1. Prediction of the traits based on polygenic risk scores in deciles among each target (twin sub-cohort) set.**

Figure interpretation: Comparing the bottom decile for the polygenic score, the top decile of polygenic score for asthma, eczema, and IBD is associated with higher odds for equivalent phenotypic traits. However, there is no clear pattern between the deciles of polygenic score for allergic rhinitis and the phenotypic measure of allergic rhinitis.


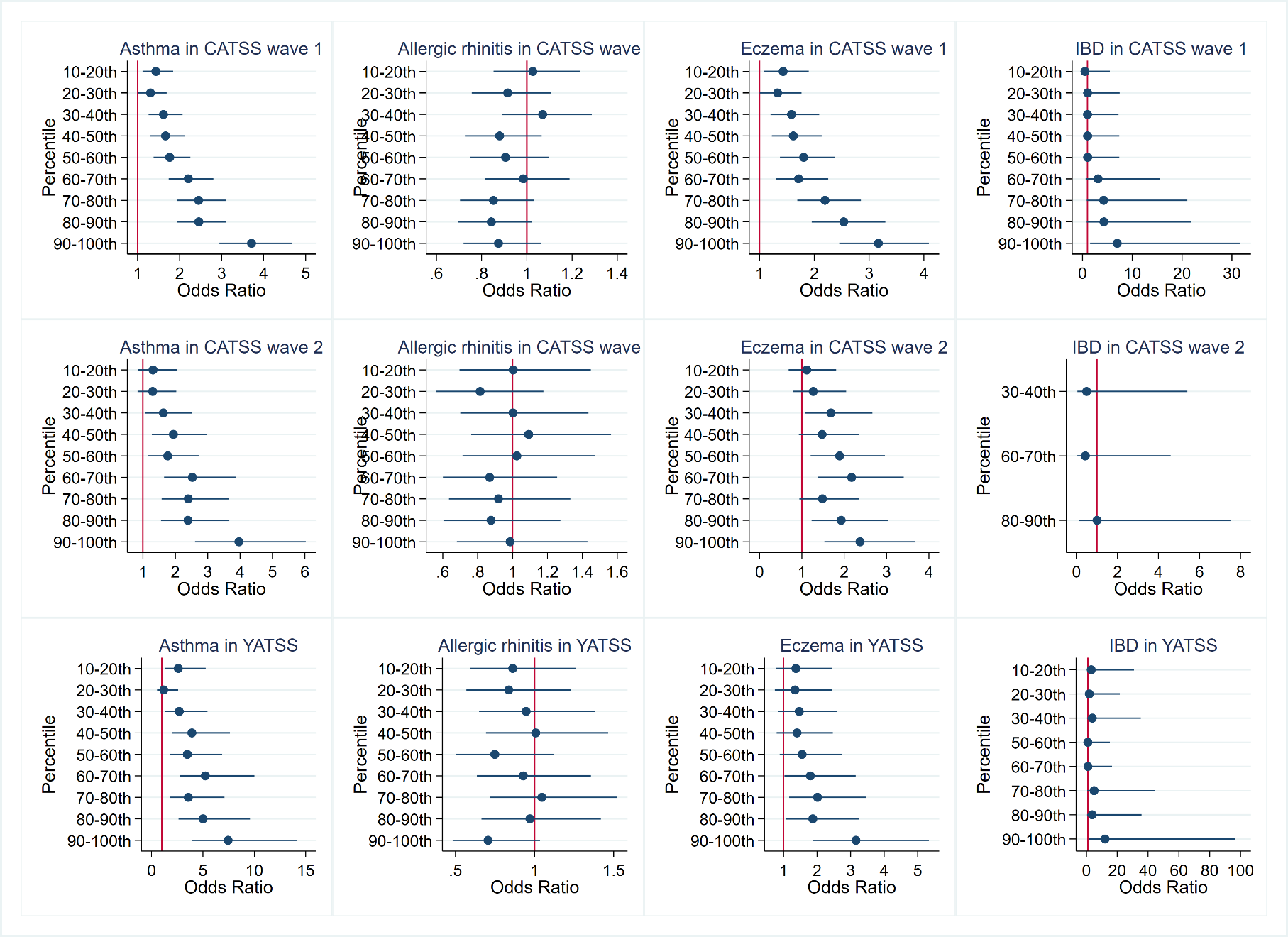


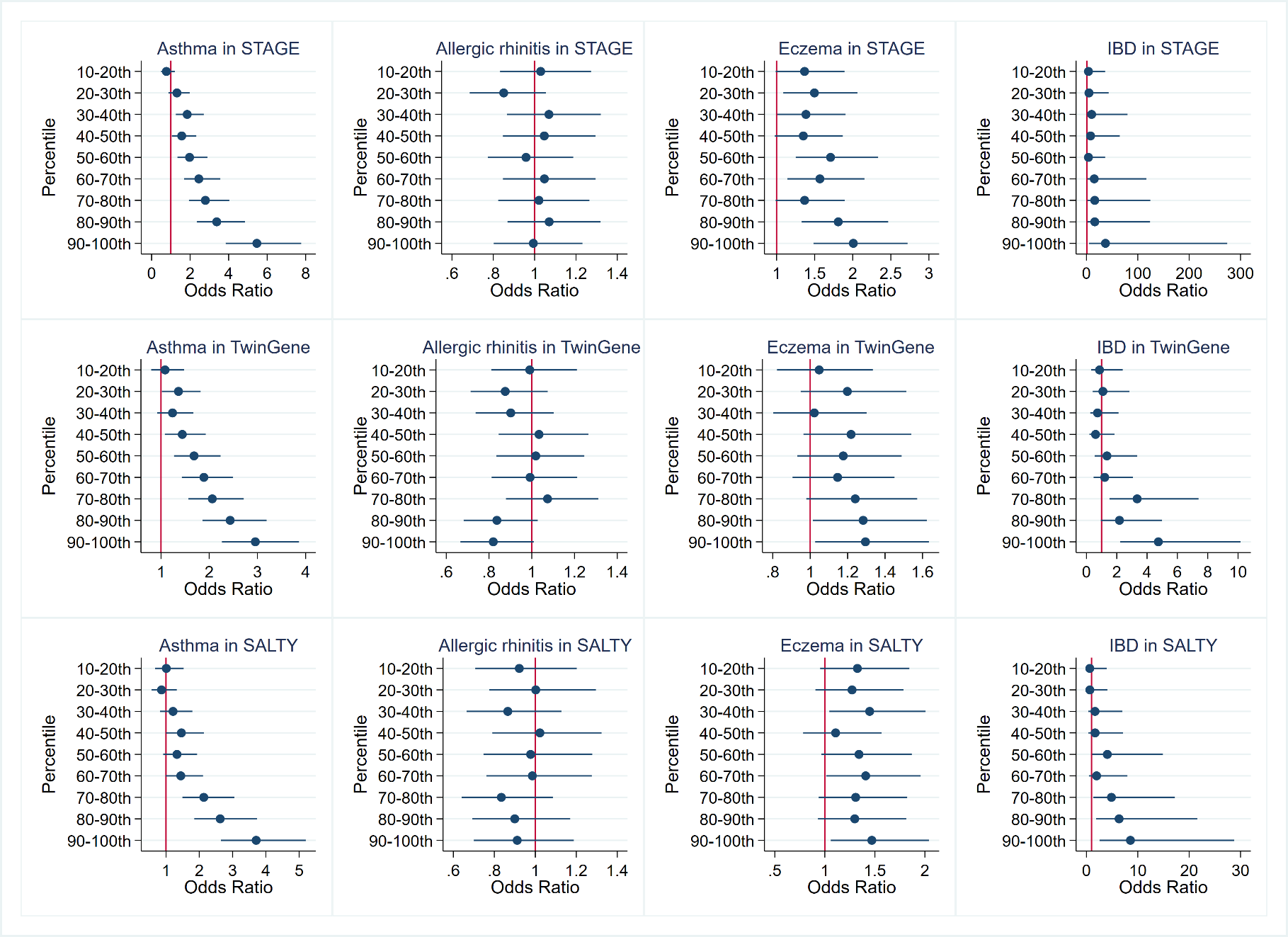


**References**

1. Zhou W, Kanai M, Wu K-HH, et al. Global Biobank Meta-analysis Initiative: Powering genetic discovery across human disease. *Cell Genomics.* 2022;2(10):100192.

2. Shrine N, Portelli MA, John C, et al. Moderate-to-severe asthma in individuals of European ancestry: a genome-wide association study. *The Lancet Respiratory Medicine.* 2019;7(1):20-34.

3. Ferreira MAR, Mathur R, Vonk JM, et al. Genetic Architectures of Childhood- and Adult-Onset Asthma Are Partly Distinct. *American journal of human genetics.* 2019;104(4):665-684.

4. Waage J, Standl M, Curtin JA, et al. Genome-wide association and HLA fine-mapping studies identify risk loci and genetic pathways underlying allergic rhinitis. *Nat Genet.* 2018;50(8):1072-1080.

5. Sliz E, Huilaja L, Pasanen A, et al. Uniting biobank resources reveals novel genetic pathways modulating susceptibility for atopic dermatitis. *The Journal of allergy and clinical immunology.* 2022;149(3):1105-1112.e1109.

6. de Lange KM, Moutsianas L, Lee JC, et al. Genome-wide association study implicates immune activation of multiple integrin genes in inflammatory bowel disease. *Nat Genet.* 2017;49(2):256-261.

7. Ortqvist AK, Lundholm C, Wettermark B, Ludvigsson JF, Ye W, Almqvist C. Validation of asthma and eczema in population-based Swedish drug and patient registers. *Pharmacoepidemiology and drug safety.* 2013;22(8):850-860.

8. Henriksen L, Simonsen J, Haerskjold A, et al. Incidence rates of atopic dermatitis, asthma, and allergic rhinoconjunctivitis in Danish and Swedish children. *J Allergy Clin Immunol.* 2015;136(2):360-366 e362.

9. Jakobsson GL, Sternegård E, Olén O, et al. Validating inflammatory bowel disease (IBD) in the Swedish National Patient Register and the Swedish Quality Register for IBD (SWIBREG). *Scandinavian Journal of Gastroenterology.* 2017;52(2):216-221.
